# Trial watch: beta-blockers in cancer therapy

**DOI:** 10.1080/2162402X.2023.2284486

**Published:** 2023-11-27

**Authors:** Killian Carnet Le Provost, Oliver Kepp, Guido Kroemer, Lucillia Bezu

**Affiliations:** a Equipe Labellisée Par La Ligue Contre Le Cancer, Université de Paris, Sorbonne Université, INSERM UMR1138, Centre de Recherche des Cordeliers, Institut Universitaire de France, Paris, France; b Metabolomics and Cell Biology Platforms, Gustave Roussy Cancer Campus, Université Paris Saclay, Villejuif, France; c Pôle de Biologie, Hôpital Européen Georges Pompidou, AP-HP, Paris, France; d Gustave Roussy, Département d’anesthésie, Chirurgie et Interventionnel, Villejuif, France

**Keywords:** Beta-adrenoreceptors, beta-blockers, cancer, catecholamines, stress

## Abstract

Compelling evidence supports the hypothesis that stress negatively impacts cancer development and prognosis. Irrespective of its physical, biological or psychological source, stress triggers a physiological response that is mediated by the hypothalamic-pituitary-adrenal axis and the sympathetic adrenal medullary axis. The resulting release of glucocorticoids and catecholamines into the systemic circulation leads to neuroendocrine and metabolic adaptations that can affect immune homeostasis and immunosurveillance, thus impairing the detection and eradication of malignant cells. Moreover, catecholamines directly act on β-adrenoreceptors present on tumor cells, thereby stimulating survival, proliferation, and migration of nascent neoplasms. Numerous preclinical studies have shown that blocking adrenergic receptors slows tumor growth, suggesting potential clinical benefits of using β-blockers in cancer therapy. Much of these positive effects of β-blockade are mediated by improved immunosurveillance. The present trial watch summarizes current knowledge from preclinical and clinical studies investigating the anticancer effects of β-blockers either as standalone agents or in combination with conventional antineoplastic treatments or immunotherapy.

## Introduction

Stress typically leads to the co-activation of the hypothalamic-pituitary-adrenal (HPA) axis and the sympathetic adrenal medullary (SAM) axis, resulting in the systemic elevation of stress hormones, namely glucocorticoids produced by the adrenal cortex and catecholamines (including epinephrine and norepinephrine) that are released from the adrenal medulla into the circulation.^1,2^ Catecholamines act on adrenergic receptors (ARs), thus increasing heart rate and blood pressure to prepare the organism for a fight-or-flight response.^3^ ARs belong to the G-protein-coupled receptor super-family and are categorized into α- and β-ARs based on their location and function. α-ARs are divided into two classes: α1-AR, predominantly found on blood vessels, which increase blood pressure upon activation, and α2-AR, mainly located within solid organs such as the pancreas, where they control insulin synthesis. β-receptors can be further classified into three types: β1-AR (found in the heart, blood vessels, kidney, and ciliary muscle), β2-AR (located in the lungs and ciliary muscle), and β3-AR (present only in smooth muscle tissue). β-ARs play a major role in stimulating cardiovascular functions and promoting the relaxation of the smooth muscles in bronchi and blood vessels. Table 1

**Table 1.** Adrenergic receptors and their main agonists and antagonists in clinic use (non-exhaustive list).

Receptors	Endogenous agonists affinity	Pharmacological agonists	Agonist properties	Pharmacological antagonists
α1	Epinephrine < norepinephrine	Epinephrine, norepinephrine, phenylephrine	Smooth muscle constriction in viscera, skin, sphincter, mucosa, vessels, iris mydriasis	Alfuzosin, hydroxyzine, tamsulosin
α2	Epinephrine = norepinephrine	Clonidine, dexmedetomidine, epinephrine, norepinephrine	Smooth muscle constriction, decreased insulin and glucagon production, decreased thyroid hormone production, decrease platelet aggregation	Diverse antipsychotics
β1	Epinephrine	Dobutamine, epinephrine, isoprenaline	Chronotropic, dromotropic, inotropic, bathmotropic effects, increased renin production	Atenolol, bisoprolol, metoprolol, nebivolol, propranolol, timolol
β2	Epinephrine > norepinephrine	Epinephrine, isoprenaline, salbutamol, terbutaline	Smooth muscle relaxation, vessels dilatation, enhanced lipolysis, bronchodilatation	Propranolol, timolol
β3	Epinephrine = norepinephrine	Amibegron, mirabegron, solabegron	Enhanced lipolysis smooth muscle relaxation of bladder and bowel	SR 59230A

β-blockers are specific antagonists targeting β-ARs. The first synthetic molecules dichloroisoprenaline and pronethalol were derived from epinephrine in the late 1950s, but were withdrawn from clinical use several years later due to severe cardiotoxic side effects.^4^ However, in 1962, propranolol, a non-cardioselective agent that targets both β1 and β2 receptors, was found to promote negative chronotropic, bathmotropic, inotropic, and dromotropic cardiac effects and a decrease in kidney renin production (resulting in decreased blood pressure), thus providing safe clinical use.^5^ Subsequently, cardioselective agents including acebutolol, atenolol, bisoprolol, and nebivolol were introduced. These agents specifically target β1-ARs, thus limiting side effects such as vaso- and bronchoconstriction. Currently, β-blockers are broadly used in clinical practice, primarily for regulating dysrhythmia, and arterial hypertension, preventing heart attacks and migraines, as well as for treating glaucoma. However, misuse of these agents can cause severe hypotension, bradycardia, asthenia, asthma or Raynaud’s syndrome, and contraindications, such as cardiac conduction disturbances or chronic obstructive pulmonary disease have to be respected. Table 1

**Figure 1.**
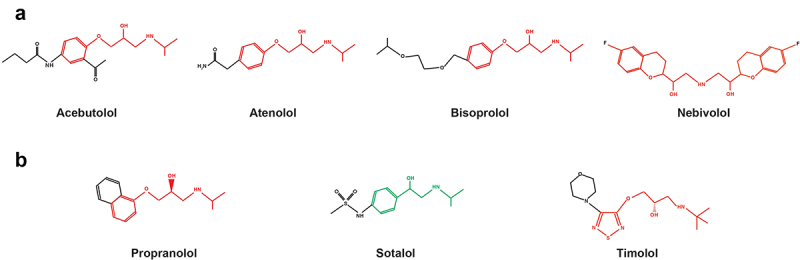
Chemical structures of the most employed cardioselective β-blockers (a) and non-selective β-blockers (b). Red = aryloxypropanolamine and green = phenylethanolamine β-blockers.

β-blockers can be subdivided into two pharmacological classes: phenylethanolamines and aryloxypropanolamines. Phenylethanolamines are composed of an aromatic group linked to an ethylamine substituted by a hydroxyl on position 1. β -blockers of this class such as sotalol or labetalol are non-cardioselective and act on-target on β1 receptors while often causing off-target β2 receptor-mediated side effects. Compounds from the group of aryloxypropanolamines including carvedilol, nebivolol and propafenone possess an aromatic group linked to a propylamine substituted by a hydroxyl on position 2 and have an additional methyl group on the amine chain, which increases their activity and selectivity. Of note, the size of the aromatic group of a β-blocker determines its ability to activate adrenergic receptors. Thus, a small aromatic group as the one of epinephrine allows activation of adrenergic receptors, whereas a voluminous group such as the one of pronethalol induces an antagonistic effect. Moreover, a β-blocker becomes cardioselective if its activity on β1 receptors is higher than that on β2 receptors. Agonistic activity on β1 receptor allows vasodilatation that decreases blood pressure, while activating β2 promotes side effects such as bronchoconstriction. This selectivity is due to a hydrogen group inducing a preferential interaction with the β1 receptor. Cardioselective β-blockers include acebutolol, atenolol, bisoprolol, and nebivolol, while the most employed non-cardioselective β-blockers are propranolol, sotalol and timolol. (Figure 1)

Surgical removal of the primary tumor plays a crucial role in improving the overall survival of cancer patients. However, the physical excision of the tumor by the surgeon can release circulating tumor cells, which can spread micrometastases to distant organs. Additionally, surgical procedures can cause stress such as pain, nociception, inflammation, and tissue damage, which in turn trigger a cascade of local and systemic signaling pathways, activating corticotropic signaling. This results in the secretion of adrenocorticotropic hormone (ACTH), catecholamines, and cortisol, proportional to the stress caused during surgery, leading to critical neuroendocrine and metabolic changes known as ‘glucocorticoid stress’.^6,7^ Moreover, stress hormones released into the systemic circulation can negatively impact both humoral and cellular immunity.^8,9^ Thus, ACTH inhibits the synthesis of immunoglobulins by plasma cells, while catecholamines can act on adrenoceptors expressed on natural killer (NK) cells, peripheral mononuclear cells and CD4^+^/8^+^ T lymphocytes,^10^ triggering the production of intracellular cyclic adenosine monophosphate (cAMP) and protein kinase A (PKA), altogether abrogating chemotaxis, migration and cytotoxic functions.^11–13^ Moreover, glucocorticoid stress can impact type I interferon (IFN) production by dendritic cells, as well as the release of IFN- γ by cytotoxic T lymphocytes (CTLs), thus broadly compromising adaptive antitumor immune responses.^14,15^ Studies in a rat mammary adenocarcinoma model also showed that injection of catecholamines inhibited NK cell-mediated tumor lysis and suppressed resistance to NK-sensitive metastasis.^16^ Furthermore, endogenous stress hormones have been shown to activate β2-ARs and downstream cAMP-PKA signaling pathways that increased the activity of matrix metalloproteinases (MMP), further promoting the dissemination of malignant cells. They were also shown to stimulate STAT3 signaling in cells surrounding the tumor tissue, leading to the release of pro-inflammatory cytokines, including IL-6 and IL-8, and an increase in the secretion of vascular endothelial growth factor (VEGF), thus promoting tumor growth and neovascularization, respectively.^17–20^ In a murine model of pancreatic cancer, chronic stress increased levels of circulating steroids and adrenal tyrosine resulting in impaired immune responses, with a decreased response of *ex vivo* splenocytes to lipopolysaccharide, a decrease in cytotoxic T-lymphocyte-associated protein 4 (CTLA-4) expression in CD4^+^ cells, and an increase in regulatory T cells in the tumor bed, altogether stimulating tumor growth and impacting on overall survival.^21^ In yet another study using a murine model of MDA-MB-231 breast cancer, stress-induced epinephrine production was found to promote tumor growth in a time- and concentration-dependent manner.^22^ Additionally, in an observational retrospective trial, Cox regression analysis revealed low serum epinephrine as a predictor of positive prognosis. Thus, breast cancer patients with low serum epinephrine levels had a significantly better overall (OS) and disease-free survival (DFS) compared to patients with high epinephrine levels.^22^ Surprisingly, colon cancer cells are also able to produce immunoregulatory glucocorticoids to suppress the activation of immune cells. Finally, many data support that both endogenous as well as exogenous corticoids might diminish therapies-induced anti-tumor response. In summary, while oncological interventions are crucial for achieving remission in clinical routine, they paradoxically decrease the immunosurveillance necessary to avoid immune escape thus promoting the development of secondary lesions due to ‘glucocorticoid stress’.^14,23,24^

Psychosocial stress has been suggested as a putative cause of cancer incidence and mortality for a long time, however, determining a cause–effect relationship has been challenging and was not firmly established.^25^ The influence of persistent corticotropic signaling on major antitumor immunological effectors, such as NK cells, dendritic cells (DC), and T-lymphocytes, has been explored through various approaches.^26^ T lymphocytes obtained from the serum of stressed individuals exhibited a shift in phenotype from Th1 to Th2, potentially affecting immune signaling pathways.^27^ Moreover, in both animal models and human studies, psychosocial stress was found to negatively impact NK cell activity and promote tumor growth. In a murine colon carcinoma model, social isolation stress decreased splenic NK cell activity while increasing angiogenesis leading to the formation of secondary tumors.^28,29^ Conversely, NK cells from ovarian cancer patients became more efficient at lysing tumor cells after receiving psychosocial support during the perioperative period.^30^ Furthermore, preclinical data suggest that psychosocial stress activates β-adrenergic signaling and promotes tumor progression. Thus, in a murine hepatocellular carcinoma model, restraint stress promoted tumor growth and increased norepinephrine levels through β-adrenergic signaling.^31^ In an orthotopic ovarian carcinoma model, mice subjected to restraint stress experienced increased tumor growth and VEGF-mediated vascularization, which correlated with the level of circulating stress hormones. However, premedication with propranolol, a β-blocker with anxiolytic properties, reversed the tumor-promoting effects.^11^ Similar results were observed in mice exposed to psychosocial stress through crowded or isolated housing conditions where stress-enhanced melanoma and fibrosarcoma growth was decreased by the oral administration of propranolol.^32^ In a social defeat model in mice; we observed stress-elevated plasma corticosterone levels and an increase in the expression of glucocorticoid-inducible factor Tsc22d3 that blocked type I Interferon (IFN) responses in dendritic cells (DC) and T cells, thus dampening therapeutic responses against carcinogen-induced and transplantable tumors. In this setting, the administration of a glucocorticoid receptor antagonist reversed the negative impact of psychosocial stress on therapeutic outcomes.^14^ Taken together, these findings suggest that effectively preventing or managing psychological stress by pharmacological strategies could significantly improve oncological prognosis. However, putative effects of psychological and medical stress on cancer induction and progression are currently discussed, and additional clinical data are expected to support this evidence and to further substantiate cause–effect relationships^14,33,34^

## Preclinical investigations

β-ARs can be found on the cell surface of different types of primary and metastatic tumor cells directly linking persistent stress with oncogenesis and disease progression.^17,35–40^ Thus, epinephrine, norepinephrine, and other AR agonists can directly act on malignant cells, leading to various pro-tumorigenic effects including enhanced proliferation and increased migratory potential.^18,35,37,39,41–43^ These stress mediators also cause intracellular hypermetabolism, as evidenced by the accumulation of lipid droplets in MCF-7 breast cancer cells treated with AR agonists *in vitro*.^44,45^ Several downstream molecular mediators have been identified as responsible for pro-tumorigenic β-AR signaling, including the intracellular second messengers cAMP and PKA, which transactivates epidermal growth factor receptor (EGFR)^46^ and triggers the initiation of the mitogen-activated protein kinase 1 (MAP2K1 better known as MEK1)/mitogen-activated protein kinase 1(MAPK1) and MAPK3 (better known as ERK1/2) cascade^47^ thus stimulating cyclin D1, cyclin E2, and cyclin-dependent kinases CDK 4/6 to promote proliferation.^17^ Moreover, β-AR signaling leads to the activation of cytosolic phospholipase-A2, the release of arachidonic acid (AA), as well as to an increased expression of the AA-metabolizing and tumor-growth-promoting enzymes cyclooxygenase-2 (COX-2) and 5-lipoxygenase in cancer cells.^48–50^ PKA also upregulates transcription factors including nuclear factor kappa B (NF-kB), activator protein 1 (AP1), and cAMP response element-binding protein (CREB) in the context of lung and pancreatic adenocarcinoma development.^51^ Additionally, β-AR activation results in enhanced retinoblastoma protein phosphorylation and suppresses Rap1B prenylation, leading to reduced cell–cell adhesion and a migratory phenotype.^35,38,42,46,52^ Interestingly, the autocrine secretion of epinephrine by certain cancer cells can stimulate β-AR, and this effect is further increased by oncogenic factors such as nicotine.^53,54^ Altogether these observations support the hypothesis that β-AR antagonists have clinical potential by halting malignant disease and decreasing the incidence of recurrences as suggested by the inverse correlation between the incidence of prostate adenocarcinoma and the use of antihypertensive drugs including β-blockers (*n* = 2442, HR = 0.7, 95% [0.5–0.9]).^55^

The exploration of different types of β-AR has been subject to extensive research. However, among cardioselective β-blockers, the nonselective propranolol appears to be particularly effective.^56^ Propranolol acts on both β1 and β2 receptors and showed the capacity to effectively mitigate oxidative stress as well as pro-tumorigenic inflammatory response such as IL-6 and TNF-α production.^57,58^ Additionally, propranolol impairs tumorigenesis in a time- and concentration-dependent manner through various molecular and cellular processes.^42,59–61^ Moreover, AR antagonists, including propranolol, have shown potential to reverse stress-induced tumorigenesis and disease progression.^19,35–39,41,52,62,63^ Thus, by blocking β-adrenergic signaling and counteracting the metastatic potential of epinephrine and norepinephrine, propranolol inhibits the invasion of malignant cells and reduces their metastatic spread to distant organs.^64^ Propranolol can intercept voltage-gated sodium channels expressed in malignant cells that are crucial for cell mobility and additionally impair the enzymes MMP2 and MMP9, which dysregulate anti-tumor immunity and facilitate the migration of malignant cells through the extracellular space.^65–67^ Moreover, AR antagonists can halt cellular proliferation through anti-mitotic effects paralyzing the cell cycle at G0/G1/S phase or G2/M phase, strongly affecting DNA synthesis.^68–76^ AR antagonists also exhibit antiangiogenic properties by decreasing the expression and activity of vascular endothelial growth factor (VEGF) thus further impacting cancer progression.^66,77,78^

Of note β-blockers can induce distinct types of cellular stress including autophagy,^79,80^ endoplasmic reticulum (ER) stress and mitochondrial dysfunction resulting in the production of reactive oxygen species (ROS) and affecting glucose metabolism,^81,82^ altogether triggering apoptotic or necro(pto)tic cell death^83–90^ of cancer cells. In addition, these stresses deeply contribute to the anti-cancer response,^91^ especially by enhancing immune infiltration of the tumor microenvironment.^92–94^ AR antagonists also exert trans-inhibitory effects on certain signaling pathways and transcription factors involved in carcinogenesis. Epidermal growth factor receptor (EGFR) is frequently overexpressed in epithelial tumors, resulting in elevated levels of intracellular cAMP and PKA, which promotes angiogenesis, invasiveness, and renders cells resistant to apoptosis. Both propranolol and atenolol can prevent the development and progression of tumors by competitively decreasing intracellular cAMP and PKA levels.^18,95^ Moreover, propranolol can arrest the cell cycle in various malignant cell types by downregulating components of the pro-invasive signaling pathways ERK/COX-2 or EGFR-Akt/ERK1–2, as well as by modifying the phosphorylation level of survival (Akt, p53, GSK3β) and mitogenic regulators (p44/42 MAPK, p38 MAPK, JNK, CREB), ultimately leading to cancer cell apoptosis.^35,47,59,68,69,96–99^ Finally, β-AR antagonists have been shown to reverse nicotine-induced mitogenic and protooncogenic factors such as COX-2, ERK1/2, PGE2, PKC, and VEGF in colon, gastric, and lung cancer cells in a dose-dependent manner.^54,100,101^

Carvedilol, yet another β- and β-AR antagonist, exerts cytotoxic effects on many human hematopoietic and solid tumor cell lines.^102^ Compared with other adrenergic agents, carvedilol exhibits a unique Ca^2+^ mobilization capacity by exerting control over extracellular Ca^2+^ influx and the release of Ca^2+^ from ER stores. The carvedilol-orchestrated increases in cytosolic Ca^2+^ movement triggers cytotoxicity and inhibits the migratory capacity of human osteosarcoma, hepatoma and oral cancer cells in a concentration-dependent manner.^85,87,103^ Carvedilol inhibits signaling pathways promoting invasiveness, including the cAMP, PKA/SCR, PKCs/Src pathways.^104^ Moreover, carvedilol inhibits EGF-mediated malignant skin transformation in a dose-dependent manner by impairing AP-1 activation.^105^ Conversely, atenolol, another β1-AR specific antagonist, failed to prevent neoplastic transformation after application to mouse epidermal cells JB6 P.^+105^

Until now β3-AR blockade was less investigated. However, an increased expression of β3-AR was reported for neuroblastoma and melanoma.^86^ Moreover, β3-ARs favor the recruitment of tumor-associated pro-inflammatory and pro-tumor effectors such as fibroblasts and M2 macrophages. The use of β3-AR antagonists, such as SR59230A and L-748 337, reportedly impacts tumor vasculature and reduces the growth of melanoma.^86,106^ SR59230A induces a significant reduction of mitochondrial activity, halting ATP synthesis and triggering the generation of reactive oxygen species, resulting in tumor cell death.^107^ Moreover, the inhibition of β3-AR reduces the proliferation of neuroblastoma by dysregulation of bioactive lipid sphingosine kinase 2/sphingosine 1-phosphate metabolism, which is implicated in various cancers and anticancer therapy resistance.^108^ Furthermore, β3-AR antagonists were shown to reduce the phosphorylation of the mTOR/p70S6K pathway, thus reducing malignant growth.^109^

β-AR antagonists have shown a significant potential in mitigating the immunosuppressive effect of chronic stress, thereby improving immunosurveillance. Thus, propranolol was shown to suppress stress-induced lung metastases in a preclinical model of murine breast cancer, while nadolol decreased the incidence of metastases promoted by surgical stress by 50%.^61,110,111^ In various models of subcutaneous cancers, propranolol prevented a stress-induced ileopathy that led to immunosuppressive dysbiosis.^112^ Propranolol also suppresses the progression of hematopoietic cancers such as acute lymphoblastic leukemia by impairing α-adrenergic signaling activated during psychological stress.^113^ Furthermore, α-blockers decrease the number of myeloid-derived suppressor cells (MDSCs). Conversely, MDSCs are induced by α2 adrenergic signaling in response to chronic stress.^114–118^

β-adrenergic receptors play an important role in shaping the immune orientation of the tumor microenvironment.^119,120^ Thus, decreasing adrenergic stress by different approaches including physiological manipulation such as placement of mice in a thermoneutral environment, genetic interventions such as the knockout of β-AR or pharmacological β-blockade, increases glycolysis and oxidative phosphorylation in tumor-infiltrating lymphocytes. Reduction of adrenergic stress upregulates the expression of the costimulatory molecule CD28, stimulates cytokine release^121^ and enhances the ration of cytotoxic over regulatory T cells. It also increases the secretion of granzyme B and IFN-γ contributing to immune-mediated anti-cancer responses in mice.^96,99,122^ Additionally, SR59230A promotes the differentiation of stromal cells and increases the abundance of lymphoid, myeloid, and NK progenitor cells in the tumor microenvironment. Altogether, these effects may inhibit tumor progression, inflammation and angiogenesis.^123^

Propranolol has demonstrated a remarkable synergism with current antineoplastic therapies. Specifically, it enhances radiosensitivity, thereby increasing radiotherapy-induced abscopal antitumor effect and exerts T cell-dependent immune response that effectively slows tumor growth. Moreover, propranolol decreases the expression of prometastatic, proinflammatory and proangiogenic genes such as EGFR, COX-2 and VEGF, respectively, thus impairing cell viability and inducing apoptosis.^60,124–127^

The cytotoxic effects of conventional chemotherapeutic agents such as platinum salts, anthracyclines (such as doxorubicin), 5-fluorouracil, mitotic spindle poisons (such as taxanes or vincristine), topoisomerase inhibitors and gemcitabine were increased in the presence of β-blocking agents including propranolol.^128–134^ β-blocking agents also boosted the anticancer effect in combination with targeted therapies such as U0126 (a MAPK inhibitor),^79^ sorafenib (a multikinase inhibitor),^80^ vemurafenib (a B-Raf gene inhibitor),^135^ and sunitinib (an inhibitor of tyrosine kinase).^136^ Importantly, propranolol was found to decrease the expression of programmed death receptor-1 (PD-1) in tumors and to increase CD8^+^ T cell infiltration within the tumor microenvironment, thus enhancing the efficacy of immune checkpoint blockade.^93,122,137–142^ If combined with the antitumor vaccine STxBE7, propranolol strongly enhanced the amount of tumor infiltrating CD8^+^ T cells, although without improving their activity.^143^

**Figure 2.**
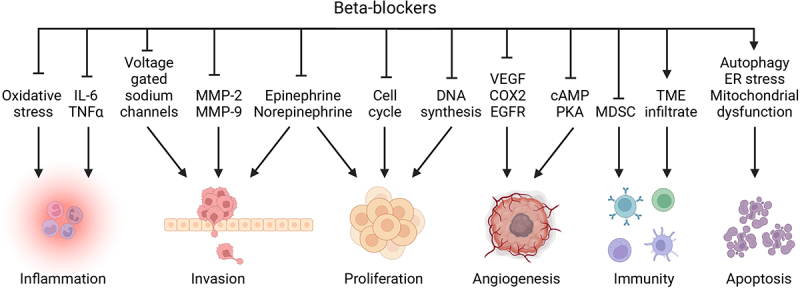
Scheme of the biological effects of β-blockers.

Even agents without any direct antitumor activity have been found to potentiate the cytotoxic effects of β-blockers. Thus, the combination of propranolol with metformin, an oral antidiabetic agent, revealed an unexpected synergistic inhibitory effect on proliferation, invasion, and migration *in vitro* and reduced tumor growth and metastasis *in vivo*.^144,145^ Similarly, when combined with the COX-2 inhibitor etodolac, propranolol increased the cytotoxicity of NK cells, reduced postsurgical local relapse and metastasis and improved overall free-survival.^146–148^ In conjunction with 2-deoxy-D-glucose (2DG), propranolol significantly reduced glucose metabolism associated with alterations in mitochondrial morphology and the subsequent activation of endoplasmic reticulum stress, autophagy, proliferative arrest ultimately resulting in apoptosis.^149^ Altogether, this underscores the potential of AR blocking agents alone or in combination with additional medication as novel anticancer (immuno)therapies. Table 2, Figure 2.

cAMP, cyclic adenosine monophosphate; COX-2, cyclooxygenase 2; EGFR, epithelial growth factor receptor; ER, endoplasmic reticulum; IL, interleukin; MDSCs, myeloid-derived suppressor cells; MMP, matrix metalloproteinase; NK, natural killer; PKA, protein kinase A; TME, tumor microenvironment; TNF, tumor necrosis factor; VEGF, vascular endothelial growth factor. Created with BioRender.com.

**Table 2.** Antitumor effects of β-blockers.

Cancer type	β-blocker	Cell lines; animal model; patient sample	Mode of action	Ref.
Angiosarcoma, hemangio-endothelioma	Propranolol	Murine angiosarcoma SVR and hemangioendothelioma EOMA cells; canine angiosarcoma Emma, Frog and SB cells	Concentration-dependent inhibition of proliferation, migration and tumor growth; apoptosis induction	^59^
Bone sarcoma	Propranolol, carvedilol	Canine osteosarcoma OSCA 32, OSCA 40 cells; human osteosarcoma HOS cells and Ewing sarcoma A673 cells	Concentration-dependent inhibition of viability; enhanced radiosensitivity	^60^
Breast cancer	Atenolol, L-748,337 (β3 antagonist)	Human breast cancer MCF-7 cells	Reduced adrenaline and isoprenaline induced lipid droplets cAMP, PKA, EPAC-dependent way	^44^
Breast cancer	Propranolol	Murine breast tumor 4T1 cells	Reduction of inducible MDSC *in vitro*; inhibition of IL-6 expression	^116^
Breast cancer, colon cancer, melanoma	Propranolol	Murine CT26.CL25, B16-F10, 4T1 cells, C57BL/6 and BALB/c mice	Potentiation of abscopal antitumor effects and T cell immune responses to radiotherapy; reduction of metastasis	^124^
Breast cancer	Propranolol	Murine breast cancer 4T1 and AT-3 cell, C57BL/6 and BALB/c mice	Reduction of MDSCs and impairment of immunosuppressive function	^114^
Breast cancer	Propranolol	Human breast cancer MDA-MB-231 cells	Inhibition of voltage-gated sodium channels, inhibition of lateral motility and invasion	^65^
Breast cancer	Propranolol, ICI 118,551 (selective β2 blocker)	Human breast cancer MDA-MB-231 cells	Inhibition of viability by downregulation of ERK, COX-2 pathways; cell cycle arrest; apoptosis	^68^
Breast cancer	Propranolol	Murine breast cancer 4T1 cells, BALB/c, NSG and C57BL/6 mice	Suppression of stress-induced lung metastasis	^110^
Breast cancer	Propranolol	Murine breast cancer 4T1 cells, BALB/c and C57BL/6 mice	Increased CTL/Treg ratio and decrease PD-1 expression in the TME; potentiation of anti-PD-1 checkpoint blockade	^122^
Breast cancer	Propranolol	Human breast cancer MDA-MB-231, IBH-6 cells	Inhibition of migration	^61^
Breast cancer	Propranolol	Human breast SK-BR-3 cells	Anti-proliferation; decreased p44/42 MAPK, p38 MAPK, JNK, and CREB signaling, increased phosphorylation of survival/apoptosis regulators AKT, p53, and GSK3β).	^97^
Breast cancer	Propranolol	Human breast *M*-406, MCF7, MDA-MB-231 cells; murine breast cancer *M*-234p, 4T1 cells; BALB/c and CBi mice	Synergistic effects with metformin, inhibition of viability, migration, invasion, mitochondrial function, tumor growth and metastasis	^144^
Breast cancer	Propranolol; CGP-20712A (β1 antagonist); ICI-118,551; L-748,337	Human breast cancer MDA-MB-231 cells; BALB/c nude mice	Inhibition of invadopodia formation (role in invasion *via* Src pathway)	^186^
Breast cancer	Propranolol	Human breast cancer MDA-MB-231 cells	Inhibition of migration	^42^
Breast cancer	Carvedilol	Human breast cancer MDA-MB-231 and MCF-7 cells	Inhibition of migration and invasion *via* inhibition of Src pathway	^104^
Breast cancer	Propranolol	Murine breast cancer 4T1 cells, human breast cancer MDA-MB-231 and MCF-7 cells	Decreased HK-2 expression; inhibition of glucose metabolism	^187^
Breast cancer	Propranolol	Human breast cancer MDA-MB-231, MDA-MB-468, MDA-MB-435S cells	Reversed norepinephrine-mediated adhesion promoted by the release of GROα and β1 integrin	^43^
Breast cancer	Propranolol	Human breast cancer MCF-7, MDA-MB-231 and SKBR3 cells, glioblastoma U87 cells, lung carcinoma A549 cells, neuroblastoma SK-N-SH cells; NMRI nude mice	Anti-proliferation (dose-dependent); anti-angiogenesis; synergistic effect *in vitro* and *in vivo* with 5-fluorouracil and paclitaxel in cell type and dose dependent manner	^128^
Breast cancer	Propranolol	Human estrogen responsive breast cancer MCF-7, ZR-75, MDA-MB-361; estrogen non-responsive breast cancer MDA-453, MDA-435, MDA-468 cells	Short exposure of MDA-453 to propranolol increases GIRK1 mRNA (potassium channel) and decrease B2-adrenergic mRNA levels	^188^
Breast cancer	Atenolol, propranolol, ICI 118,551	Human estrogen responsive breast cancer MCF-7, ZR-75, MDA-MB-361; estrogen non-responsive breast cancer MDA-453, MDA-435, MDA-468 cells	Inhibition of DNA synthesis	^76^
Cervical cancer	Propranolol	Human cervical carcinoma Siha, HeLa cells	Anti-proliferation by suppressing cGMP/PKG pathway; inhibition of clone formation; apoptosis induction	^83^
Colorectal cancer	Propranolol	Human colorectal cancer HCT116, HT29, RKO cells	Apoptosis induction; decreased mitochondria and proteins involved in oxidative phosphorylation; reduced metastatic potential, viability and proliferation	^81^
Colorectal cancer	Propranolol	Murine colorectal cancer CT26 cells; BALB/c mice	Decreased tumor growth by activating CD8^+^ T cells and by inhibiting AKT/MAPK pathway	^96^
Colorectal cancer	Atenolol, carvedilol, propranolol, ICI 118,551	Human colorectal cancer HT-29 cells	Reversion of AR agonists-induced proliferation	^62^
Colorectal cancer	Propranolol	Murine colorectal cancer CT26 cells; BALB/c mice	Improved resistance to metastasis in combination with etodolac	^146^
Colorectal cancer	Atenolol, propranolol, ICI 118,551	Human colorectal cancer SW1116, SW480, SW620, DLD1, HCT116, Colo205, HT29 cells, BALB/c mice	Inhibition of viability and tumor growth (G1-phase cell cycle arrest and inhibition of EGFR-Akt/ERK1/2 pathway), apoptosis induction	^69^
Colorectal cancer	Atenolol, ICI 118,551	Human colorectal cancer HT-29 cells	Dose-dependent inhibition of nicotine-induced tumor growth, microvessel densities; expression of COX2, PGE2, VEGF	^100^
Colorectal cancer	Atenolol, propranolol	Human colorectal cancer SW 480 cells	Inhibition of norepinephrine-promoted migration	^41^
Cutaneous squamous-cell carcinoma	Butoxamine (β2 antagonist), ICI 118,551	Human skin epidermoid carcinoma A-431 cells	Inhibition of VEGF-A-induced angiogenesis; decreased tumor development	^77^
Gastric cancer	Propranolol	Human gastric cancer MKN45, NUGC3 cells	Inhibition of tumor growth, viability (G1 cycle arrest), and migration; apoptosis induction; decreased expression of P-CREB-ATF and MEK-ERK pathways; suppression of MMP2, MMP9 and VEGF expression	^70^
Gastric cancer	Propranolol ICI 118,551	Human gastric cancer HGC27, MKN45, MGC803, BGC823, SGC7901, AGS cells	G1/S phase cell cycle arrest; apoptosis; inhibition of tumor growth, proliferation, invasion, and metastasis by inhibiting ERK1/2-JNK-MAPK pathway and transcription factors (NFkB, CREB, STAT3)	^71^
Gastric cancer	Propranolol	Human gastric cancer SGC7901l cells; nude mice	Potentiation of radiotherapy effects, decreased tumor growth, decreased expression of NFkB and then COX-2, EGFR, VEGF expression)	^125^
Gastric cancer	ICI 118,551	Human gastric cancer NCI-N87, MGC803, HGC27, BCG823 cells	Decreased catecholamine-stimulated MUC4 expression involved in the resistance of trastuzumab in HER2^+^ gastric cancer	^189^
Gastric cancer	Propranolol	Human gastric cancer SGC7901, BCG823 cells	Inhibition of proliferation (G0/G1 arrest and G2/M arrest) in a concentration-dependent manner; apoptosis; decreased expression of NFkB, VEGF, COX-2, MMP2, MMP9	^72^
Gastric cancer	Propranolol	Human gastric cancer SGC7901, BCG823 cells	Potentiation of radiation effects, decreased viability and clonogenic potential, apoptosis through the inhibition of NFkB/VEGF/EGFR/COX-2 pathways	^126^
Gastric cancer	Atenolol, ICI 118,551	Human gastric cancer AGS cells	Reversion of nicotine-induced expression of PKC, ERK1/2 phosphorylation, and COX-2 with cell proliferation	^100^
Glioma	Propranolol	C6 rat glioma	Inhibition of TNFα-induced proliferation	^190^
Kidney cancer	Propranolol, ICI 118,551	Patient derived ccRCC cells; human renal cell carcinoma 786-O cells	Decreased viability; decreased oxidative stress and mRNA expression of proinflammatory cytokines	^58^
Kidney cancer	Propranolol, ICI 118,551	Human renal cell carcinoma 786-O cells; NSG mice	Apoptosis induction; inhibited expression of HIF2α, CAIX, VEGF; impaired nuclear internalization of HIF2α and NFkB/p65, reduced tumor growth	^84^
Leukemia	Propranolol	Human leukemia Molt-4, Jurkat, U937 cells	Decreased VEGF and MMP2 activity	^66^
Leukemia	SR59230A (β3 antagonist)	Human myeloid leukemia K562, KCL22, HEL, HL60 cells	Apoptosis induction, doxorubicin-resistance reversion	^123^
Leukemia	Propranolol	Human pre-B acute lymphoblastic leukemia Nalm-6 cells; NCID mice	Reversed stress effect of by decreasing tumor burden and dissemination	^113^
Liver cancer	Propranolol	Murine hepatocellular carcinoma H22 cells; BALB/c mice	Inhibition of tumor growth induced by chronic stress and inactivation of CXCL5-CXCR2-ERK pathway	^191^
Liver cancer	Propranolol	Murine hepatocellular carcinoma H22 cells	Inhibition of tumor growth; prevention of the redistribution of splenic myeloid cells	^31^
Liver cancer	Propranolol	Human liver cancer HepG2, HepG2.2.15, HL-7702 cells	Anti-proliferation; apoptosis induction; S-phase cycle arrest	^73^
Liver cancer	Carvedilol	Wistar rats with hepatic cirrhosis	Decreased hepatocarcinogenesis by suppression of circulating IL-6, ALT, AST, ALP, Bilm, and hepatic IL-6, STAT-3, MDA levels and hydroxyproline content	^192^
Liver cancer	Carvedilol	Human hepatoma HA59T cells	Increased intracellular calcium; apoptosis	^85^
Liver cancer	ICI 118,551	HCC cell lines, C57BL/6 mice	Autophagy; HIF1α destabilization; tumor growth suppression; improved anti-tumor activity of sorafenib	^80^
Lung cancer	Atenolol, betaxolol, esmolol, metoprolol, pindolol, propranolol, timolol	Human lung carcinoma A549, H1299 cells	Apoptosis, necrosis induction; inhibition of lung cancer cell colony formation	^90^
Lung cancer	ICI 118,551	Human NSCC lung cancer UMSCC103, Cal33 cells	Inhibition of p38 and NFkB oncogenic pathways; affect ERK and PI3K pathways, Nrf2-Keap1 stability and its nuclear translocation; induction of ROS and oxidative stress; synergistic effect with U0126 (MAPK inhibitor) on viability and induction of autophagy	^79^
Lung cancer	Propranolol	Patient-derived head and neck cancer cells	Inhibition of proliferation and viability	^39^
Lung cancer	Esmolol, ICI 118,511, nadolol	Human lung cancer A549, MRC-5 cells	Apoptosis ROS induction	^82^
Lung cancer	Propranolol	Rat breast cancer MADB106 cells; F344 rats	Combined with etodolac (COX-2 inhibitor): reduction of tumor retention induced by surgery, increase NK cytotoxicity	^147^
Lung cancer	Nadolol	Rat breast cancer MADB106 cells; F344 rats	Decrease surgery-induced metastasis	^111^
Melanoma	Propranolol, ICI 118,551	Murine melanoma B16F10 cells	Inhibition of AR agonist induced proliferation	^176^
Melanoma	SR59230A	Murine melanoma B16F10 cells	Decreased tumor growth, proliferation and viability; induced differentiation of stromal cells in the TME, promote hematopoietic differentiation; increased number of NK cells	^129^
Melanoma	Propranolol	Human melanoma MEL270, OMM2.5, MP41, MP46, WM115, WM266.4 cells	Anti-proliferation; anti-migration; VEGF reduction; cell cycle arrest; apoptosis	^78^
Melanoma	SR59230A	Human melanoma A375 cells	Reversion of reduction of mitochondrial activity mediated by β3-AR/UCP2 axis	^107^
Melanoma	Propranolol	Human melanoma A375 cells; patient derived- melanoma cells; NOD/SCID mice	Synergistic effect with sunitinib (anti-proliferation, cell cycle arrest through suppressing ERK/Cyclin D1/Rb/Cyclin E, decrease tumor growth)	^136^
Melanoma	Propranolol	MT/ret mice	Decreased angiogenesis, proliferation and survival; decreased infiltration of immunosuppressive myeloid cells in TME	^115^
Melanoma	Propranolol	Human melanoma A375 cells, patient derived melanoma cells; BALB/c mice	Cell cycle arrest; apoptosis *via* AKT/MAPK pathway	^98^
Melanoma	L 748,337	Murine melanoma B16F10 cells	Decreased tumor growth, neoangiogenesis and proliferation; increased tumor cell death	^106^
Melanoma	Propranolol	Patient derived melanoma cells	Anti-proliferation; anti-angiogenesis; apoptosis; decreased tumor growth	^56^
Melanoma	SR59230A L 748,337	Murine melanoma B16F10 cells	Anti-proliferation; apoptosis; decreased tumor growth	^86^
Melanoma	Propranolol	Human melanoma A375, Hs29-4T cells	Decrease mobility and released IL6/VEGF induced by catecholamines	^17^
Melanoma	Metoprolol, Propranolol, ICI 118 551	Murine melanoma B16F10 cells	Potentiation of anti-PD-1 checkpoint blockade	^137^
Multiple myeloma	Bisoprolol, Propranolol ICI 118 551	Human multiple myeloma LP-1, OPM-2, RPMI-8226, ANBL-6, XG-2 cells	Apoptosis; autophagy; mitochondrial respiratory chain alteration; decreased glycolysis; increased chemosensitivity to melphalan and bortezomib	^193^
Neuroblastoma	Propranolol	Human neuroblastoma KELLY, CHLA-20, LAN-5, IMR-32, SK-N-BE1, SK-N-BE^2^, SK-N-BE^2^c, SK-N-SH, SK-N-AS, LAN-6, SH-EP, CHLA-15, CHLA-90, SK-N-FI cells	Inhibition of growth and proliferation; apoptosis *via* activation of p53 and p73; synergistic effect with SN-38 (topoisomerase I inhibitor)	^130^
Neuroblastoma	Atenolol, butoxamine, carvedilol, labetalol, metoprolol nebivolol, propranolol	Human neuroblastoma BE^2^-C, SHEP, SK-N-SH cells	Synergistic effect with vincristine (anti-angiogenic, anti-mitochondrial, anti-mitotic, pro-apoptotic effects, decrease tumor growth)	^131^
Neuroblastoma	SR59230A	Human neuroblastoma SK-N-BE^2^ and BE^2^C cells; murine neuroblastoma Neuro-2A cells; NCI A/JCr mice	Decreased proliferation; inhibition of tumor growth and progression through blockade of SK2/S1P_2_ signaling	^108^
Neuroblastoma	SR59230A	Neuroblastoma cell lines from patients	Anti-proliferation; Inhibition of tumor growth and colony formation through the inactivation of the mTOR/p70S6K pathway	^109^
Osteosarcoma	Carvedilol	Human osteosarcoma MG63 cells	Anti-proliferation Increase in intracellular calcium level, which may lead to cytotoxicity	^103^
Osteosarcoma	Propranolol	Human osteosarcoma MG63, U2OS cells	Decreased tumor growth; anti-mitotic; G0/G1 cycle arrest; impaired colony formation, 3D spheroid growth, cell chemotaxis and capillary-like tube formation; alteration of cytoskeleton	^74^
OSCC	Propranolol	Human tongue cancer SCC-9, SCC-25, Cal27 cells	Dose- and time-dependent decrease in viability; downregulated p-P65 NFkB and VEGF expression; inhibited cell migration; synergism with CDDP and 5-FU	^132^
OSCC	Propranolol	OSCC cells; BALB/c mice	Reversed anti-migratory effect induced by AR agonist	^63^
OSCC	Propranolol	4NQO induced oral carcinogenesis in Wistar rats	Decreased occurrence of tumors; decreased thickness of OSCC; reduced pro-inflammatory cytokines IL-6 and TNF-alpha	^57^
OSCC	Propranolol	Human tongue cancer SCC-9, SCC-25 cells	Reversion of migratory effect of norepinephrine	^36^
OSCC	Carvedilol	Human oral cancer OC2 cells	Apoptosis	^87^
OSCC	Propranolol	Human tongue cancer TCa8113 cells; human salivary adenoid cystic carcinoma ACC cells	Reversed migratory and mitogenic effect of norepinephrine	^37^
Esophagus cancer	Atenolol, ICI 118,551	Human esophageal squamous cell carcinoma HKESC-1 cells	Reversion of proliferative effect of epinephrine	^18^
Esophagus cancer	Atenolol, ICI 118,551	Human esophageal squamous cell carcinoma HKESC-1 cells	Inhibition of proliferative effects mediated by epidermal growth factor	^38^
Ovary cancer	Propranolol	Murine ovarian cancer HeyA8, SKOV3ip1, A2780, RMG-II, MB-231 cells	Reversion of AR agonist and daily stress-induced tumor growth and angiogenesis.	^11^
Pancreas cancer	ICI 118,551, metoprolol	Human pancreatic adenocarcinoma MIA PaCa-2, BxPC-3 cells	G1/S phase cell cycle arrest; apoptosis; anti-proliferative, anti-invasive effect	^75^
Pancreas cancer	Atenolol, propranolol, ICI 118,551	Pancreatic ductal adenocarcinoma patient cohort	Synergistic effects with gemcitabine on survival	^133^
Pancreas cancer	Propranolol	Human pancreatic carcinoma Panc-1 cells	Reversion of pro-tumor effects of norepinephrine	^35^
Pancreas cancer	Propranolol	Human pancreatic cancer Colo-357, BxPC-3, MIA PaCa-2, Panc-1, PaTus cells; murine pancreatic cancer 6606PDA, TD1, TD2, Panc02 cells; C57BL/6 mice	Reversion of catecholamine-induced proliferation and migration	^21^
Pancreas cancer	ICI 118,551	Human pancreatic cancer BxPC-3, MIA PaCa-2 cells	Synergistic effect with gemcitabine (anti-proliferative and pro-apoptotic effects); inhibition of NFkB	^134^
Pancreas cancer	Metoprolol, propranolol, ICI 118,551	Human pancreatic cancer BxPC-3, MIA PaCa-2 cells	Anti-proliferative and anti-invasive effect by inhibition of cAMP/PKA and Ras, NFkB, AP-1, CREB, VEGF, MMP2, MMP9, COX-2	^67^
Pancreas cancer	Propranolol	Human pancreatic cancer BxPC-3, MIA PaCa-2 cells	Reversion of the invasive effect of norepinephrine (concentration-dependent)	^19^
Pancreas cancer	Propranolol	Nitrosamine-induced pancreas cancer in Syrian golden hamsters	Anti-tumor effect by blocking cAMP-dependent intracellular signaling, cAMP-dependent release of EGF and PKA-dependent release of VEGF	^95^
Pancreas cancer	Butoxamine, metoprolol, propranolol	Human pancreatic adenocarcinoma PC-2 cells	Apoptosis	^88^
Pancreas cancer	Propranolol	Human pancreatic adenocarcinoma Panc-1 cells	Reversion of the pro-tumor effect (tumor growth) of AR agonist	^52^
Pancreas cancer	Propranolol	Human pancreatic adenocarcinoma Panc-1, HPAF-II, Capan-1 cells; BALB/c-Foxn1nu mice	Reversion of proliferative and invasive effects induced by the activation of AR by chronic stress	^23^
Prostate cancer	Propranolol	Human pancreatic adenocarcinoma PC-3 cells	Propranolol + 2DG (glycolysis inhibitor) induced autophagy, anti-proliferative effects, apoptosis, mitochondrial morphology alteration, endoplasmic reticulum stress, and suppression of tumor growth	^149^
Prostate cancer	Propranolol	Human pancreatic adenocarcinoma PC-3 cells; BALB/c mice	Reversion of norepinephrine-induced lumbar lymph metastasis	^64^
Prostate cancer	ICI 118,551	Human prostate cancer C42 cells	Prevention of stress-induced effects	^194^
Bladder cancer, prostate cancer	Propranolol	Human prostate cancer C4, human prostate cancer, human urinary bladder carcinoma T24 cells	Apoptosis; inhibition of MAPK pathway	^89^
Thyroid cancer	Propranolol	Human thyroid cancer K1, BCPAP, ATC, BHP27 cells	Inhibition of tumor growth; apoptosis; cell cycle arrest; decreased expression HK2 and GLUT1; Synergism with vemurafenib	^135^
Uveal melanoma	Carvedilol	Human uveal melanoma Mel270, 92–1, UPMD2, UPMM3 cells	Inhibition of tumor growth; prevention of long-term survival; additive cytotoxic effect with radiation	^127^
Colorectal cancer, melanoma	Propranolol	Murine melanoma B16-OVA cells, colorectal cancer CT26.CL25 cells; C57BL/6NCr and BALB/c mice	Decreased tumor growth in stressed mice; reduced checkpoint receptors; increased glycolysis/mitochondrial dysfunction, oxidative phosphorylation and CD28 expression in TILs; increased expression of anti-tumor cytokines	^121^
Lung cancer, skin cancer	Carvedilol	Murine epidermal cancer JB6 P^+^ cells; human lung cancer A549 cells	Inhibition of EGF-induced malignant transformation; inhibition of EGF-mediated activator protein-1 (AP-1) activation	^105^
Breast cancer, Colorectal cancer, liver cancer	Atenolol, propranolol, ICI 118,551	Human breast cancer MCF7, colorectal cancer HT29, liver cancer HepG2 cells	Anti-proliferative, anti-migratory and anti-invasive effect	^40^
Lung cancer, melanoma	Propranolol	Murine melanoma B16F10.9, Lewis lung carcinoma 3LL cells; C57BL/6J mice	Combined with COX-2 inhibitor etodolac: improved overall survival and recurrence free-survival; reversed surgical glucocorticoid stress: increase NK cytotoxicity and expression of Fas ligand and CD11a; decreased corticosterone level	^148^
Breast cancer, cervix adenocarcinoma, melanoma, leukemia	Carvedilol	Human breast cancer MDA-MB-231; melanoma Fem-x; cervix adenocarcinoma HeLa, leukemia K562 cells	Anti-proliferative effect	^102^
Fibrosarcoma, melanoma	Propranolol	Murine melanoma B16, fibrosarcoma Meth A cells; C57BL/6 and BALB/c mice	Inhibit psychological stress-enhanced tumor growth	^32^
/	Atenolol, propranolol, ICI 118,551	Preclinical *in vivo* model (xenografts)	Inhibition of cell viability and xenograft growth; decreased phosphorylation of AKT/MEK/ERK; activation of CD8^+^ T cells in TME	^99^
/	Propranolol	Mouse model of vaccine-based immunotherapy (C57BL/6J and OT-IxSJL) + TC1 expressing HPV16-E6/E7	Improved efficacy of antitumor STxBE7 vaccine by enhanced the CD8^+^ T cell infiltration	^143^

## Published clinical trials

The aforementioned preclinical findings spurred the initiation of numerous clinical trials most of which are observational (12 retrospective trials, 1 prospective trial and 6 meta-analyses). Retrospective studies come from cohorts of oncological patients treated with β-blockers for a history of cardiovascular disease or arterial hypertension or from data extracted of prospective trials with incidental use of β-blockers. Most of the results concluded to a positive impact of β-blockers on oncological outcomes. β-blockers significantly decreased tumor growth and the risk of metastasis into distant organs. As a result, most analyses reported a strong positive correlation between the use of β-blockers and overall and progression free-survival in various treated solid tumors.^150–161^ These positive and encouraging results need to be confirmed in prospective randomized controlled trials. Indeed, these studies involved many sources of bias such as combination of antihypertensive drugs (β-blockers plus angiotensin-II-receptor inhibitor or angiotensin-converting enzyme inhibitors) and positive effects of β-blockers on cardiovascular mortality. Only three trials failed to report prognostic effects of β-blockers^162–164^ except a better response to pembrolizumab in the treatment of stage III melanoma.^162^ Only one study reported negative effects of β-blockers in a cohort of HER2+ breast cancer patients treated with trastuzumab, where β-blocker appeared to decrease survival (PFS adjusted HR = 2.21, 95%[1.56–3.12]; *p* < 0.001 and OS adjusted HR = 2.46, 95%[1.69–3.57]; *p* < 0.001)^165^. However, the higher rate of mortality observed in this trial might involve cardiovascular mortality or immune toxicity. Table 3

**Table 3.** Published observational studies (prospective, retrospective, and meta-analyses) investigating the inhibition of adrenergic signaling pathway in cancer patients.

Cancer	Beta-blockers	Conventional anticancer agents	Results	Study	Ref.
Brain cancer	β-blockers	NA	225 patients included. Control of tumor growth (*p*=0.001), tumor progression (*p*=0.0001) and higher survival (*p*=0.015). Strong correlation between β-blockers and survival (*p*=0.049)	Retrospective	^150^
Breast cancer (HER2+)	Atenolol, bisoprolol, carvedilol, propranolol	Trastuzumab	PFS adjusted HR=2.21, 95%CI[1.56–3.12]; *p*<0.001 OS adjusted HR=2.46, 95% CI[1.69–3.57]; *p*<0.001	Retrospective	^165^
Breast cancer (HER2-)	Atenolol, bisoprolol, metoprolol, propranolol	Docetaxel and/or ramucirumab	Improved PFS (15.5 *vs* 8.3 months); *p*=0.038 No significant difference in OS	Retrospective	^151^
Breast cancer	Atenolol, bisoprolol, propranolol, timolol	NA	Reduction in metastasis (*p*=0.026), tumor recurrence (*p*=0.001) and longer disease-free interval (*p*=0.01). A 57% reduction in risk of metastasis (HR=0.430; 95%CI[0.200–0.926], *p*=0.031). A 71% reduction in mortality after 10 years (HR=0.291; 95%CI[0.119–0.715]; *p*=0.007). No significant difference in vascular invasion.	Retrospective	^152^
Breast cancer	β-blockers	NA	46 245 patients included. Survival HR=0.44; 95%CI[0.26–0.73] with I^2^=78%. DFS HR=0.71; 95%CI[0.19–1.03]	Meta-analysis	^195^
Colo-rectal cancer	β-blockers	Chemotherapy (2628 patients) Radiotherapy (1427 patients)	4794 patients included. β-blockers decreased mortality (adjusted OR=0.88; 95%CI[0.77–1.00]; *p*=0.04)	Retrospective	^153^
Hepato-cellular carcinoma	Carvedilol, nadolol, propranolol, timolol	NA	47 studies (28 RCT + 19 cohorts) included. No significant association between propranolol (OR=0.94;95%CI [0.62–1.44]) or timolol (OR=1.32; 95%CI [0.44–3.95]) and HCC incidence. Risk of HCC decreased by 26% and 38% with nadolol (OR=0.74; 95%CI[0.64–0.86]; *p*=0.796) and carvedilol (OR=0.62; 95%CI[0.52–0.74]; *p*=0.776).	Meta-analysis	^154^
Hepato-cellular carcinoma	Carvedilol, propranolol, nadolol, timolol	NA	23 studies were included (totaling 2618 patients). β-blockers do not reduce mortality.	Meta-analysis	^196^
Lung cancer	Landiolol	Adjuvant chemotherapy (8 patients)	28 patients included in the landiolol group and 29 in the control group. HR for RFS in the landiolol group was 0.41; 95%CI[0.13–1.34]; *p* = 0.1294. HR for RFS in the landiolol group without adjuvant chemotherapy was 0.50; 95%CI[0.15–1.62]; *p* = 0.2363.	Retrospective	^197^
Lung cancer	Selective agents: atenolol, bisoprolol, metoprolol Non selective agents: carvedilol, labetolol, nadolol, propranolol, sotalol	Radiotherapy (100% patients) Chemotherapy (90% patients)	722 patients (155 patients received β-blockers). Univariate analysis: better Distant Metastasis Free Survival (*p*<0.01), DFS (*p*<0.01), and OS (*p*=0.01) compared with no β-blockers. Multivariate analysis: better DMFS (HR=0.67; *p*=0.01), DFS (HR=0.74; *p*=0.02), and OS (HR=0.78; *p*=0.02)	Retrospective	^155^
Melanoma	β-blockers	NA	121 patients (30 patients treated with β-blockers). A 36% risk reduction of progression each year in the treated group (95%CI[11%-54%]; *p*=0.002)	Retrospective	^156^
Melanoma	Selective agents: acebutolol, atenolol, betaxolol, bisoprolol, celiprolol, esmolol, metoprolol, nebivolol Non-selective agents: carteolol, carvedilol, labetatol, levobunolol, metipranolol, nadolol, oxprenolol, penbutolol, pindolol, practolol, propranolol, sotalol, timolol	± pembrolizumab	No prognostic effect of β-blockers on RFS (HR=0.67; 95%CI [0.38–1.19] in the pembrolizumab group and HR=1.15; 95%CI [0.80–1.66] in the placebo group).	Retrospective	^164^
Ovary cancer	Atenolol, bisoprolol, metoprolol, oxprenolol, pindolol, propranolol	NA	Extension of at least 8 years post-surgery if use non selective β-blockers	Retrospective	^157^
Ovary cancer	Metoprolol	No chemotherapy (9 patients) IV platinum (146 patients) IV-IP platinum (30 patients)	Metoprolol given before and during cytoreduction for 70 patients *vs* 115 patients (control group). OS was significantly higher in β-blocker group (44.2 *vs* 39.3 months; *p*=0.01). In multivariate analysis, β-blocker was associated with significant improvement in OS (HR 0.68; 95%CI[0.46–0.99]; *p*=0.046).	Retrospective	^158^
Ovary cancer	Acebutolol, atenolol, betaxolol, bisoprolol, metoprolol, nebivolol, penbutolol, propranolol, talinolol, soltalol	carboplatin, gemcitabine	No difference in PFS (7.79 *vs* 7.62 months; *p*=0.95) and OS (21.2 *vs* 17.3 months; *p*=0.18) between β-blockers group and control group.	Retrospective	^163^
Prostate cancer	β-blockers	No hormono- radio- or chemotherapy prior surgery (exclusion criteria)	11 117 men were included. β-blockers at time of surgery were significantly associated with a lower risk of treatment for cancer recurrence (adjusted HR=0.64; 95% CI[0.42–0.96]; *p*=0.03)	Prospective	^159^
Solid tumors	Atenolol, bisoprolol, carvedilol, labetalol, metoprolol, nebivolol, solatol	Immunotherapy (PD-1, PD-L1, CTLA-4 inhibitors) ± chemotherapy	11 studies included (=10 156 patients). No association between β-blockers and OS (HR=0.97; 95%CI[0.85–1.11]) or PFS (HR=0.98; 95%CI[0.90–1.06]). Significant better response to immunotherapy in the cohort (OR=0.42; 95%CI[0.19–0.94]; *p*=0.036) and lung cancer subgroup (OR= 0.25; 95%CI [0.08–0.83]); *p*=0.024)	Meta-analysis	^162^
Solid tumors	Acebutolol, atenolol, bisoprolol, carvedilol, celiprolol, labetolol, metoprolol, nadolol, nebivolol, oxprenolol, pindolol, propranolol, timolol	NA	Reduction in risk of cancer (HR=0.33 95%CI[0.13; 0.83]; *p*=0.019). In the meta-analysis sub-analysis: lower risk of cancer (MH-OR=0.93 95%CI[0.86; 1.01]; *p*=0.070).	Observational and meta-analysis	^160^
Solid tumors	β-blockers	Chemotherapy and radiotherapy in some studies	20 cohorts and 4 case controls (76 538 patients). β-blockers were given after the diagnosis of cancer. HR all causes mortality=0.89 (95%CI [0.81–0.98]). HR cancer mortality=0.89 (95%CI [0.79–0.99]).	Meta-analysis	^161^
Solid tumors	β-blockers	Immunotherapy (PD-1, PD-L1, CTLA-4 inhibitors)	13 studies included (=3 331 patients). Concomitant use of NSAIDs, β-blockers and metformin is not associated with improved OS or PFS.	Meta-analysis	^198^

**Abbreviations**: CI, confidence interval; DMFS, distant metastasis free survival; HR, hazard ratio; IV, intravenous; IP, intraperitoneal; NSAIDs, non-steroidal anti-inflammatory drugs; OS, overall survival; PFS, progression free-survival; RFS, recurrence free-survival.

Nine prospective interventional or randomized controlled trials have been published. One study enrolled 25 participants with multiple myeloma to receive either propranolol in titrated doses or placebo for 5 weeks. This trial concluded on the tolerability and the efficacy of propranolol to minimize β-adrenergic stress during hematopoietic cell transplantation. It also showed successful engraftment and effective response against myeloma when propranolol was administered.^166^ Safety and efficacy of β-blockers on adrenergic stress during the treatment of 26 ovary cancers were also confirmed.^167^ Used as a standalone agent during breast cancer and melanoma care, propranolol increases immune cell infiltration into the tumor bed and significantly reduces the risk of recurrence.^168,169^ When associated with conventional antineoplastic treatments such as taxanes or anthracyclines, β-blockers are also perfectly tolerable and safe.^170^ Moreover, propranolol potentiates the immune effects of pembrolizumab against melanoma.^171^ Combined with anti-COX-2 during the peri-operative period of breast and colorectal cancer surgery in three randomized controlled trials, propranolol minimizes surgical stress, improves tumor molecular markers, reinforces the anti-tumor immune response, impairs pro-inflammatory and metastatic transcription factors and finally decreases the risk of relapse.^172–175^

Finally, a few prospective studies all reported promising oncological outcomes. Thus, the study of Ramondetta *et al*. showed that patients with ovary cancer treated with propranolol had 55.5% complete response, 33.3% partial response, 5.6% stable response, and only 5.6% progressive disease^165^. In the randomized controlled trial by De Giorgi *et al*. the administration of β-blocker to patients with melanoma decreased the risk of recurrence (80%, HR = 0.18, 95% CI [0.04–0.89], *p* = 0.03) with a DFS 89% *vs* 64% (*p* = 0.04). Treatment with β-blocker also decreased the rate of progressive disease (15.8% *vs* 41.2%) and death (10.5% *vs* 17.7%).^167^ Associated with pembrolizumab, β-blockers allowed to achieve 7 partial responses and 1 stable disease in a cohort of 9 metastatic melanoma patients.^169^ Of note, four studies notified cardiovascular events such as hypotension and bradycardia due to the consumption of β-blockers without major consequences.^164,166,168,169^ Table 4

**Table 4.** Published interventional and randomized controlled trials investigating the inhibition of adrenergic signaling pathway in cancer patients.

Cancer	Beta-blockers	Conventional anticancer treatment	Results	Cardiovascular effects	Study	Phase	Ref.
Breast cancer (HER2+)	Propranolol	Neoadjuvant chemotherapy (taxanes/ anthracyclines)	10 patients included. Propranolol started at 20mg and then increased to 80mg daily. Feasibility of combining propranolol with chemotherapy.	Bradycardia in 3 patients	Interventional	II	^170^
Breast cancer	Propranolol	No neoadjuvant chemotherapy (exclusion criteria)	60 patients randomized to receive placebo or propranolol (escalating doses 80-160mg daily for 7 days before surgery). Reduction in intratumoral mesenchymal polarization and increase in immune cell infiltration.	Minimal reduction in blood pressure (<20 mmHg) and heart rate (<10 mmHg) Bradycardia in 1 patient Hypotension in 3 patients	RCT	II	^168^
Breast cancer	Propranolol + etodolac	NA	38 patients randomized to receive propranolol + etodolac 5 days before and 6 days after surgery or placebo. Significant decrease in epithelial to mesenchymal transition, prometastatic/proinflammatory transcription factors and tumor-infiltrating monocytes. Increase in tumor-infiltrating B cells. Abrogation of serum IL-6 and C-reactive protein levels, and IL-12/IFNγ production.	NA	RCT	II	^172^
Breast cancer	Propranolol	With or without parecoxib No neoadjuvant chemo- or radiotherapy (exclusion criteria)	101 women were randomized to receive propranolol ± parecoxib or placebo before and after mastectomy. β-blockers reduced surgical stress-induced Treg cells. No additive or synergic effect with parecoxib.	Propranolol group: significant decrease in heart rate during the per-operative period	RCT	NA	^173^
Colo-rectal cancer	Propranolol + etodolac	NA	34 patients included to receive propranolol + etodolac for 5 days before and 15 days after surgery or placebo. Significant improvement (*p*<0.05) of tumor molecular markers. In compliant patients group, recurrence rates were 0% in the treatment group and 29.4% in the placebo group (*p*=0.054).	NA	RCT	II	^174^
Melanoma	Propranolol	Pembrolizumab	9 patients included in three groups (10 mg, 20 mg or 30 mg propranolol twice a day). Association with immunotherapy was safe, tolerable, increased IFN-γ and decreased IL-6. 10 mg group: 2 partial responses and 1 stable disease 20 mg group: 2 partial responses and 1 progressive disease 30 mg group: 3 partial responses	Hypotension in 1 patient (30 mg)	Interventional	I	^171^
Melanoma	Propranolol	NA	53 patients included (19 received propranolol). β-blockers are associated with a 80% risk reduction in recurrence (HR=0.18; 95%CI [0.04–0.89]; *p*=0.03). Propranolol group 15.8% disease progression and 10.5% death vs 41.2% and 17.7% in the control group. DFS 89% propranolol group vs 64% at 3 years, *p*=0.04). Cox model : HR DFS 0.18, 95%CI [0.04–0.89], *p*=0.03; HR OS 0.64, 95%CI [0.1–3.96], *p*=0.63	NA	RCT	NA	^169^
Multiple myeloma	Propanolol	Melphalan	25 patients included. Feasibility to recruit and treat multiple myeloma during hematopoietic cell transplantation.	Hypotension in 1 patient	RCT	II	^166^
Ovary cancer	Propranolol	Carboplatin and paclitaxel	26 patients included. Feasibility of propranolol before chemotherapy or surgery. Decrease in adrenergic stress markers. 55.5% complete response/33.3% partial response/5.6% stable response/5.6% progressive response	NA	Interventional	I	^167^

**Abbreviations**: DFS, disease free-survival; HR, hazard ratio; IFN, interferon; IL, interleukin; RCT, randomized controlled trial.

## Completed clinical trials

The primary focus of these trials was to study the effect of β-blockers as standalone agents. Among these trials, NCT01544959 evaluated the possibility of substituting fentanyl with esmolol for anesthesia induction, followed by metoprolol during mastectomy, with the aim to manage hemodynamic, perioperative pain and postoperative nausea and vomiting. NCT02596867, a non-randomized phase II trial, studied the effect of β-adrenergic blockade with 0.75 mg/kg propranolol, administered twice daily for 3 weeks, prior to surgical resection of breast cancer. The trial assessed the tumor proliferative index using Ki-67 before and 3 weeks after propranolol administration and assessed the capacity of propranolol to decrease tumor proliferation in breast cancer. Similarly, the early phase I study NCT02013492 evaluated the potential of propranolol administered for 4 weeks in patients with locally-recurrent or metastatic solid tumors to decrease tumor growth by inhibiting the effects of adrenergic hormones on the tumor cells. Furthermore, the study NCT03861598 aimed at establishing a correlation between circulating tumor cells and favorable magnetic resonance imaging (MRI) results in patients with grade IV glioblastoma receiving chemotherapy with a combination of escalated doses of carvedilol. Further trials investigated whether the synergistic effects observed between β-blockers and conventional chemotherapies observed in preclinical studies were assessable in clinical practice. Both NCT01308944 and NCT01504126 aimed at confirming feasibility of administering β-blockers before, during and after surgical ovarian cancer debulking until completion of chemotherapy to mitigate depression and anxiety and to evaluate an impact on immune response and survival. The randomized multicenter phase 2 study NCT01857817 was designed to evaluate clinical benefits and changes in circulating prostate-specific antigen (PSA) of the VT-122 protocol consisting of a co-administration of 22 mg propranolol plus etodolac 340 mg in clinically progressive prostate cancer. Table 5

**Table 5.** Completed and terminated clinical trials investigating the inhibition of adrenergic signaling pathway in cancer patients (not yet published).

Cancer	Drug	Assessed outcomes	Status	Phase	Co-therapy	Study	NCT
Breast cancer	Esmolol Metoprolol	–Postoperative consumption of narcotic –Impact of postoperative acute and chronic pain –Nausea, vomiting –Recurrence	Completed	NA	As a single agent	RCT	NCT01544959
Breast cancer	Propanolol	–Decrease in tumor proliferative index (Ki-67)	Terminated	II	As a single agent	Interventional	NCT02596867
Breast cancer	Propranolol	–Number of patients with pathologic complete response at 6 months	Completed	II	Combined with neoadjuvant chemotherapy	Interventional	NCT01847001
Gliobla-stoma	Carvedilol	–Correlation between RT-qPCR assay for circulating tumor cells and the change in response to MRI results –Evaluate response	Terminated	Early phase I	Combined with standard chemotherapy	Interventional	NCT03861598
Ovary cancer	Propanolol	–Feasibility of concurrent β–blocker administration with chemotherapy –DFS –Blood markers –Immunohistochemistry of angiogenic markers on tumor samples	Completed	I	Concurrent chemotherapy	Interventional	NCT01308944
Ovary cancer	Propanolol	–Complete 6 cycles of chemotherapy –Change in mood state –DFS and OS –Change in immune response (IL-6, IL-8, VEGF)	Completed	Early phase I	Combined with chemotherapy	Interventional	NCT01504126
Prostate cancer	Propanolol	–Change in PSA –Pain –Time to symptom progression –Change in correlative biomarkers	Terminated	II	Combined with etodolac	RCT	NCT01857817
Solid tumors	Propanolol	–Toxicity –Change in VEGF –Effect of β-adrenergic blockade on the TME and on the host immune system –DFS and OS	Completed	Early Phase I	As a single agent	Interventional	NCT02013492

Abbreviations: CRS, cytokine release syndrome; DFS, disease-free-survival; IL, Interleukin; MRI, magnetic resonance imaging; ORR, overall response rate; OS, overall survival; PSA, prostate specific antigen; RCT, randomized controlled trial; TME, tumor microenvironment; VEGF, Vascular Endothelial Growth Factor.

## Ongoing clinical trials

Most registered currently ongoing studies aim at evaluating the clinical impact, in particular overall survival and disease-free survival, after administration of β-blockers during the management of various solid tumors and hematological malignancies. Some of these trials also investigate potential advantages arising from the combination of β-blockers with additional anticancer or non-anticancer agents.

### β-blockers as single agents

The randomized placebo-controlled clinical trial MELABLOCK (NCT02962947) is designed to evaluate the efficacy and safety of a daily dose of 80 mg propranolol in patients with stage II/IIIA melanoma. NCT04518124 consists of a single arm propranolol administered in a dose of 40–80 mg 2–3 times per day immediately after diagnosis of cutaneous angiosarcoma extending over a period of 3–6 weeks. Clinical and histological responses defined as a decrease in Ki-67 index will be measured until study completion. The early phase I interventional trial NCT03245554 intends to enroll in total 80 patients with gastric adenocarcinoma or non-metastasized colon cancer without any prior treatment. Propranolol will be administered for 1 week as a neoadjuvant treatment during the preoperative period and CT-scan and Ki-67 index will be used to evaluate tumor growth. NCT02944201 aims to investigate whether β-blockers administered to prostate cancer patients from the point of diagnosis until prostatectomy can decrease tumor growth measured by Ki-67 index and TUNEL assay performed on prostatectomy tissues. Notably, the phase II study NCT05679193 presents as a large randomized controlled trial that aims to assess the potential of a 3 weeks propranolol regimen in reducing recurrences after robot-assisted laparoscopic prostatectomy, a surgical procedure significantly less inflammatory and stressful as compared to conventional laparotomy. The study will assess changes in catecholamines and PSA levels during the perioperative period, the bioavailability of propranolol as well as the impact on anesthesiological and surgical strategies including the use of vasopressors, and the occurrence of complications. Finally, NCT05312255 focuses on the evaluation of immune effects, stress reduction and quality of life in non-chemotherapeutic interventions such as physical activity, specific nutritional regimen and propranolol treatment in patients with multiple myeloma.

### β-blockers combined with conventional anticancer therapies

Four interventional clinical trials are planned to examine safety and efficacy of propranolol administered in combination with conventional chemotherapy to potentiate antitumor responses. NCT04005365 aims at incorporating propranolol into neoadjuvant chemotherapy in gastric cancer patients with the aim to improve overall response. NCT02641314 aims at administering a combination of propranolol with NSAID (celecoxib) anti-neuroblastic drugs (cyclophosphamide, etoposide and vinblastine) in children and adolescents with recurrent or progressive neuroblastoma. The primary objective is to demonstrate the non-inferiority in survival compared with controls, while as secondary outcomes safety, tolerance, and disease response rate will be assessed. NCT03108300 aims at assessing overall and progression-free survival in patients with metastatic soft tissue sarcoma co-administered propranolol 40 mg twice daily and doxorubicin. NCT02897986 is a dose escalation trial to determine the maximal tolerated dose of vinorelbine administered in combination with daily oral propranolol in children and teenagers with refractory solid tumors. NCT04682158 aims to determine the safety and efficacy of propranolol combined with standard neoadjuvant chemoradiation, as well as an impact on overall survival and pathologic response rate in patients undergoing esophageal cancer resection. The randomized phase II clinical trial NCT04493489 focuses on safety and efficacy of propranolol in adjuvant BCG therapy after the transurethral resection of bladder cancer and evaluates the capacity of the combination treatment to decrease relapses over a 2-year period. Table 6

**Table 6.** Ongoing clinical trials investigating the inhibition of adrenergic signaling pathway in cancer patients.

Cancer	Drug	Outcomes	Status	Phase	Co-therapy	Study	NCT
Angio-sarcoma	Propanolol	–Clinical and histological response (decrease of >30% of Ki-67 index)	Recruiting	II	As a single agent	Interventional	NCT04518124
Bladder	Propanolol	–Two-year recurrence-free survival	Not yet recruiting	II	Combined with BCG vaccine	RCT	NCT04493489
Breast	Propanolol	–Cytotoxic activity of NK cells, levels of NKT cells, lymphocytes, monocytes and granulocytes; cytokine levels; *In vitro* cytokine secretion; levels of cortisol and VEGF. −5-year recurrence	Unknown	NA	Combined with etodolac during perioperative period	RCT	NCT00502684
Colo-rectal	Propanolol	−5-year DFS –Tumor-infiltrating leucocytes. Pro-tumor and inflammatory cytokines –Adverse effects (Clavien-Dindo classification, depression, anxiety, global distress, fatigue)	Recruiting	II	Combined with etodolac during perioperative period	RCT	NCT03919461
Colo-rectal	Propanolol	–Recurrence –Magnitude and duration of surgically induced immune depression –Morbidity. Mortality	Unknown	III	Combined with etodolac during perioperative period	RCT	NCT00888797
Esophagus	Propanolol	−5-year DFS and OS.	Recruiting	II	Combined with chemoradiation	RCT	NCT04682158
Gastro-esophagus	Propanolol	–Toxicities and adverse events –DFS. ORR. OS.	Not yet recruiting	II	Combined with pembrolizumab, fluorouracil, oxaliplatin and leucovorin	Interventional	NCT05651594
Gastric	Propanolol	–ORR	Unknown	II	Combined with neochemo-therapy	Interventional	NCT04005365
Gastro-intestinal	Propanolol	–Tumor size (Computed tomography and Ki-67)	Unknown	Early phase I	As a single agent	Interventional	NCT03245554
Liver	Propanolol	–Failure free survival –Clinical benefit response	Unknown	II	Combined with etodolac and sorafenib	RCT	NCT01265576
Liver, pancreas, gallbladder	Propanolol	–Efficacy in boosting the effects of immunotherapy –Feasibility. Safety –Tolerability –DFS. OS	Not yet recruiting	II	Combined with durvalumab, gemcitabine, paclitaxel, tremelimumab, cisplatin	Interventional	NCT05451043
Lung	Landiolol	−2-year relapse-free survival and OS after surgery –additional treatment after recurrence. Safety events. Postoperative complications	Unknown	III	As a single agent	RCT	No NCT PMID31829248
Melanoma	Propanolol	–Dose limiting toxicities –DFS. ORR. OS.	Recruiting	I/II	Combined with pembrolizumab	Interventional	NCT03384836
Melanoma	Propanolol	–DFS. OS. Mortality –Long-term safety	Unknown	II/III	As a single agent	RCT	NCT02962947
Melanoma	Propranolol	–PFS. ORR. OS	Not yet recruiting	I	Combined with ipilimumab and nivolumab	Interventional	NCT05968690
Multiple myeloma	Propanolol	–Changes in immune cell subsets –Changes in the gut microbiome –Comparison in bone markers –Changes in body composition –Changes in stress, anxiety, fatigue, functional status, nutritional behavior before and after intermittent fasting and stress-related biomarkers	Recruiting	NA	As a single agent	Interventional	NCT05312255
Neuro-blastoma	Propanolol	–Event free survival –Disease control rate at 6 and 12 months –OS –Hospitalization days. –Number of transfusion days –Drop-out rate	Recruiting	II	Combined with celecoxib, cyclo-phosphamide, vinblastine, etoposide	Interventional	NCT02641314
Pancreas	β-blockers	–DFS. OS.	Recruiting	NA	As a single agent or combined with aspirin, metformin, ACE-inhibitors, statins	Observational Prospective	NCT04245644
Pancreas	Propanolol	–Recurrence –Tumor-infiltrating leukocytes. Protumor and inflammatory cytokines –Adverse effects (Clavien-Dindo classification, depression, anxiety, global distress, fatigue)	Recruiting	II	Combined with etodolac	RCT	NCT03838029
Prostate	Carvedilol	–Change in biomarkers (Ki-67, TUNEL assay) in prostate biopsy –Change in serum PSA	Not yet recruiting	II	As a single agent prior to surgery	Interventional	NCT02944201
Prostate	Propanolol	–Feasibility –Safety and tolerability –Bioavailability –Changes in catecholamines levels in perioperative period. Serum level of PSA –Change in distress –Surgical complications Proportion of patients requiring vasopressors. Procedure time. Blood loss. –Tumor-infiltrating leucocytes. –Prognostic and predictive markers	Recruiting	II	As a single agent	RCT	NCT05679193
Sarcoma	Propanolol	–DFS. OS	Unknown	II	Combined with doxorubicin	Interventional	NCT03108300
Solid tumors Hematolo-gical malignancy	Metoprolol	–Safety and tolerability –Efficacy for CRS control and precaution	Recruiting	I/II	Combined with CAR-T cells therapy ± infliximab, etanercept, tocilizumab and/or other agents	Interventional	NCT04082910
Solid tumors	Propanolol	–Decrease in toxicity of chemotherapy	Unknown	I	Combined with vinorelbine	Interventional	NCT02897986
Urothelial	Propanolol	–DFS. ORR. OS. –Adverse events	Recruiting	II	Combined with pembrolizumab	Interventional	NCT04848519

**Abbreviations**: ACE, angiotensin-converting enzyme; BCG, Bacillus Calmette-Guérin; CAR-T cells, chimeric antigenic receptor-T cells; CRS, cytokine release syndrome; DFS, disease-free survival; NK, natural killer; ORR, overall response rate; OS, overall survival; PFS, progression free survival; PSA, prostate specific antigen; RCT, randomized controlled trial; TUNEL, terminal deoxynucleotidyl transferase dUTP nick end labeling.

### β-blockers combined with immunotherapy

Five interventional trials (NCT05651594, NCT05451043, NCT03384836, NCT04848519, NCT01265576, NCT05968690) aim at exploring the addition of β-AR antagonists to immunotherapy (with pembrolizumab, durvalumab, tremelimumab, ipilimumab or nivolumab) alone or together with standard chemotherapy in the treatment of unresectable advanced and/or metastatic digestive adenocarcinoma, stage III/IV melanoma and urothelial cancers. The primary objective is to evaluate the efficacy of β-blockade in boosting immunotherapy as measured by response evaluation criteria in solid tumors (RECIST). Secondary explorative objectives encompass correlations of biomarkers (such as immune effectors, circulating cytokines and stress) with efficacy, progression-free survival and overall survival.

NCT04082910 will test the β1 adrenergic receptor blocker metropolol for its ability to control and prevent of cytokine release syndrome (CRS) in patients with lymphoma and leukemia enrolled for chimeric antigen receptor (CAR)-T cell infusion. Safety and tolerability of metropolol will be confirmed by evaluating heart rate and blood pressure, while the efficacy in the control of CRS will be assessed by monitoring body temperature and serum IL-6 levels.

### β-blockers combined with other agents

Four prospective trials (NCT00502684, NCT03919461, NCT00888797, NCT03838029) aim at combining β-blockers with COX-2 inhibitors before, during, and after breast or colorectal cancer surgery to evaluate the impact on immune effector activity and tumor-infiltrating leucocytes. Moreover, cytokine secretion and serum levels of cortisol and VEGF will be assessed alongside psychological examinations. Oncological outcomes such as morbidity and mortality will be assessed in a 5-year follow up. Lastly, the monocentric observational prospective study NCT04245644 will evaluate if the combination of β-blockers with four chemopreventive agents (angiotensin converting enzyme inhibitors, aspirin, metformin, and statin) can improve the overall and disease-free survival in 800 patients with pancreatic ductal adenocarcinoma.

## Discussion

Stress-related hormones and signaling *via* β-adrenergic receptors impact oncogenesis. Preclinical researches and pilot clinical trials support the hypothesis that β-blockers administered at clinically relevant concentrations during anticancer therapy extend the lifespan of individuals with malignant disease. Whether this effect is due to the direct blockage of β-AR and/or due to an indirect systemic effect *via* the reduction of stress and corticotropic activity is still under discussion. Indeed, upon physical, biological, and psychological stress, the corticotropic HPA axis is activated releasing substantial amounts of immunosuppressive catecholamines and cortisol. Catecholamines are ligands of β-AR located at the plasma membrane of cytolytic immune effectors, negatively impacting mobility and activity, but also on β-AR present on cancer cells where they can stimulate proliferation, invasion, and migration. Of note, different β-AR subtypes are present with a strong predominance of the β2 sub-group.^18^ ICI 118 551, the most selective β2-AR antagonist, demonstrated consistent antitumor activity while no or partial effects were observed with the β1-specific blocker atenolol.^41,105^ The implication of β1-receptors is still uncertain, and further investigation is required to understand the contribution of each type of receptor in tumor development. Selective β3-AR antagonists have been even less studied, however, preclinical data noticed antiproliferative and apoptotic effects, suggesting β3-AR could be a target for anticancer therapy.

At present, in the field of oncology, preference is giving to nonspecific β-blockers such as propranolol, which targets both β1/2 ARs. However, it is important to consider potential side effects arising from dual β1/2 blocking. Due to the expression of β-ARs at the tumor cell surface, some research has explored the use of carvedilol, a α-blocker, with alpha-blocking property and has demonstrated an anti-tumor effect. Thus, reduced tumor cell proliferation was observed following the use of α2-AR agonists such as clonidine, a common anti-hypertensive agent.^176^ This might indicate a crosstalk between α2- and β2-ARs in stimulating cancer cell growth. Further research is needed to elucidate the pro- or anti-tumor profiles of α-ARs.

An interesting aspect was advanced with clinical trial NCT01544959 in which authors hypothesized that fentanyl, an opioid used to control acute pain during induction and maintenance of anesthesia, could be replaced by β-blockers. Opioids have been ascribed with pro-tumorigenic effects, such as stimulating tumor cell migration by the activation of MMPs, activation of the oncogene c-MYC and inhibition of anti-angiogenic TSP-1.^177–179^ Alternative techniques such as “opioid-free-anesthesia”, a specific protocol mixing local anesthetics, ketamine, magnesium and dexmedetomidine, are emerging for the management of acute pain during the per-operative period. In this setting, due to their anti-tumor effect and immunostimulatory properties, β-blockers appear as an appealing option for opioid replacement. Reportedly, β-adrenergic stimulation inhibits the primary phase of CD8^+^ T lymphocytes activation, thus providing a rationale for the administration of β-blockers together with T cell targeted immunotherapies.^122^

Surgical glucocorticoid stress is significantly activated by local and systemic inflammatory pain that can be caused by surgical tissue damage and is mediated by pro-tumorigenic COX-2. Several randomized controlled trials advanced the possibility to potentiate antitumor effects by blocking sympathetic signaling and managing pain with a combination of β-AR antagonists and COX-2 inhibitors (NCT00502584; NCT03919461; NCT00888797; NCT03838029). COX-2 inhibitors belong to the family of non-steroidal anti-inflammatory drugs (NSAIDs) and previous observational clinical trials supported the notion that NSAIDs might improve oncological outcomes. Thus, Desmedt *et al*. observed that intraoperative administration of ketorolac, a COX-1 and COX-2 inhibitor, significantly reduced the incidence of distant recurrences after breast cancer surgery.^180^ In a study involving 327 women undergoing mastectomy, Forget *et al*. reported a significantly lower recurrence rate (*p* = 0.019) after administration of ketorolac prior to surgery, while other analgesics, such as sufentanil, ketamine and clonidine, showed no effect.^181^ A retrospective analysis in a cohort of 720 breast cancer patients revealed that injection of the NSAIDs ketorolac and diclofenac during conservative breast cancer surgery correlated with improved disease-free (HR = 0.57, *p* = 0.01) and overall survival (HR = 0.35, *p* = 0.03).^182^ Finally, in a propensity score matching study involving 2502 patients with non-small cell lung cancer, immunotherapy combined with the administration of NSAIDs was associated with a better overall survival (HR = 0.85, *p* < 0.001).^183^ *In vitro*, ketorolac was found to decrease proliferation, migration and angiogenesis, and to enhance the sensitivity of renal cancer to apoptosis. In mice, ketorolac caused tumor growth inhibition when used as a standalone agent or combined with sunitinib.^184^ In a model of head and neck squamous cell carcinoma, COX-2 inhibition showed additive and synergistic effects with EGFR inhibitors enhancing tumor cell death both *in vitro* and *in vivo*, especially in PIK3CA-mutated cancers.^99^ Altogether, the combination of COX-2 inhibitors with β-blockers seems to be a promising option to potentiate antitumor responses.

In addition, activation of β-ARs in response to surgical stress promotes the survival of circulating tumor cells and increases the incidence of metastases. Surgical glucocorticoid stress can be optimally managed by spinal anesthesia or epidural anesthesia during the perioperative period.^185^ By controlling stress and inflammatory pain, medullar anesthesia also positively impacted oncological outcome by decreasing the incidence of recurrence. However, these analgesic procedures are contraindicated in bleeding disorders, infection, backbone osteosynthesis or upon patient refusal, necessitating alternative analgesia techniques. In this case, anxiolytic premedication (the day before and 2 h before surgery), for instance with benzodiazepines, can control surgical stress. Premedication has four main objectives: i) decreasing the level of anxiety and its physical consequences such as tachycardia, perspiration and polypnea; ii) minimizing the intensity of biochemical reactions and metabolic activity; iii) enhancing the analgesic potency of anesthetics; and iv) blocking parasympathetic effects induced by anesthesia and surgery such as salivary hypersecretion, nausea, vomiting, laryngeal spasms, and dysrhythmia. Here, β-blockers offer an appealing premedication option because they address all the aforementioned objectives without the known side effects of benzodiazepines such as sleepiness, confusion, and balance disorder. In contrast to other cardiologic agents targeting the angiotensin system, β-blockers are not contraindicated prior to anesthetic procedures. To reverse or inhibit the surgical glucocorticoid stress-induced immune system suppression, β-blockers used at a dose not triggering hypotension, could be considered for cancer patients, especially in the case of contraindication for medullar anesthesia. So far, specific guidelines for the use of β-blockers during the preoperative period are still lacking, and the efficacy of such premedications still requires further demonstration.

## Conclusion

All forms of stress, be they physical, biological, or of psychological origin, can activate sympathetic signaling pathways promoting the release of catecholamines and other stress mediators into the systemic circulation. Catecholamines promote the proliferation, migration, and invasion of tumor cells, while suppressing the cytolytic function of immune effectors, through direct interaction with β-AR located on their plasma membrane. β-blockers can counteract such deleterious effects by competitively interfering with the binding of catecholamines to AR. Promising results from preclinical and clinical pilot studies suggesting that β-blockade can improve the outcome of cancer treatments now await confirmation in ongoing prospective randomized controlled trials. The facts that β-blockers are already approved and widely used for cardiovascular indications may accelerate their deployment in oncological practice.

## Abbreviations

AA: arachidonic acid
ACE: angiotensin-converting enzyme
ACTH: adrenocorticotropic hormone
AP-1: activator protein 1
AR: adrenergic receptor
BCG: Bacillus Calmette-Guérin
cAMP: cyclic adenosine monophosphate
CAR-T cells: chimeric antigenic receptor-T cells
ccRCC: clear cell Renal Cell Carcinoma
CDDP: cisplatin
CI: confidence interval
COX-2: cyclooxygenase 2
CREB: cAMP response element-binding protein
CRS: cytokine release syndrome
CTLA4: cytotoxic T-lymphocyte-associated protein 4
CTLs: cytotoxic T lymphocytes
DC: dendritic cell
DFS: disease-free survival
EGF: epidermal growth factor
EGFR: epithelial growth factor receptor
ER: endoplasmic reticulum
ERK: extracellular signal-regulated kinase
5-FU: 5-fluorouracil
HPA: hypothalamic-pituitary-adrenal
HR: Hazard ratio
HPA: hypothalamic-pituitary-adrenal
IFN: interferon
IL: interleukin
MAPK: mitogen activated protein kinase
MDSCs: myeloid-derived suppressor cells
MMP: matrix metalloproteinase
NFkB: nuclear factor kappa B
NK: natural killer
NSAIDs: non-steroidal anti-inflammatory drugs
ORR: overall response rate
OS: overall survival
OSCC: oral squamous cell carcinoma
PD-1: programmed death receptor 1
PFS: progression-free survival
PGE2: prostaglandin E2
PKA/C: protein kinase A/C
PSA: prostate-specific antigen
RCT: randomized controlled trial
RFS: recurrence-free survival
ROS: reactive oxygen species
TME: tumor microenvironment
TUNEL: terminal deoxynucleotidyl transferase dUTP nick end labeling
VEGF: vascular endothelial growth factor
VHL: Von Hippel Lindau
